# The radiofrequency interference on CRT‐D functioning during AV node ablation: An educational case

**DOI:** 10.1002/joa3.12467

**Published:** 2020-11-22

**Authors:** Giacomo Mugnai, Andrea Volpiana, Stefano Cavedon, Alessandro Mecenero, Davide Ambroso, Cosimo Perrone, Claudio Bilato

**Affiliations:** ^1^ Division of Cardiology Arzignano Hospital Arzignano (Vicenza) Italy; ^2^ TECSAL SNC Sarcedo (Vicenza) Italy

**Keywords:** AV nodal ablation, interference, radiofrequency

## Abstract

We have described some unusual findings of radiofrequency interference with ICD functioning during AV nodal ablation, guiding the reader to the possible explanation of the phenomena.

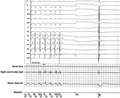

We describe the case of ablation of the atrioventricular (AV) node in a patient with a cardiac resynchronization therapy defibrillator (CRT‐D). The device (Inogen X4 G148—Boston Scientific) was previously implanted because of dilated cardiomyopathy with severely depressed left ventricular ejection fraction (LVEF), with the high voltage lead placed in the middle septum of the right ventricle. The right ventricular lead was an Endotak Reliance SG 0293 (Boston Scientific) and the left ventricular lead was an Acuity X4 Spiral L4677 (Boston Scientific). The threshold of the right ventricular lead was 0.4 V@0.4 ms before the ablation and remained unchanged after the procedure.

The percentage of stimulation in biventricular mode resulted poor (about 21% of the total electrical activity) because of atrial fibrillation with high ventricular rate despite optimal medical therapy: the patient, therefore, underwent AV nodal ablation.

Before the procedure CRT‐D tachyarrhythmia detection was disabled; VVI pacing frequency was lowered from 70 to 30 beats per minute. Through the right femoral vein, a single, nonirrigated ablation catheter (Blazer 8 mm; Boston Scientific) was positioned in the Hisian region in order to record the His signal. Then, the catheter was mildly withdrawn toward the atrium in order to place it in the fast pathway area.

Once the radiofrequency (RF) application was delivered at 70 W and 55°C of temperature, a fast junctional tachycardia started and within a few seconds the complete AV block was obtained. Although the device had been programed in VVI mode at 30 bpm, a long (>3 seconds) pause was observed after the last beat of the tachycardia, followed by a paced beat partially captured by the myocardium (Figure 1). RF application was then interrupted and the device resumed normal stimulation in VVI mode at 30 bpm. The ablation was definitively carried out, after reprogramming the device in VOO mode: complete AV block was obtained with an escape junctional rhythm at 35 bpm. The pacing output of right ventricular lead was 3.0 V@0.4 ms during the ablation procedure.

**FIGURE 1:**
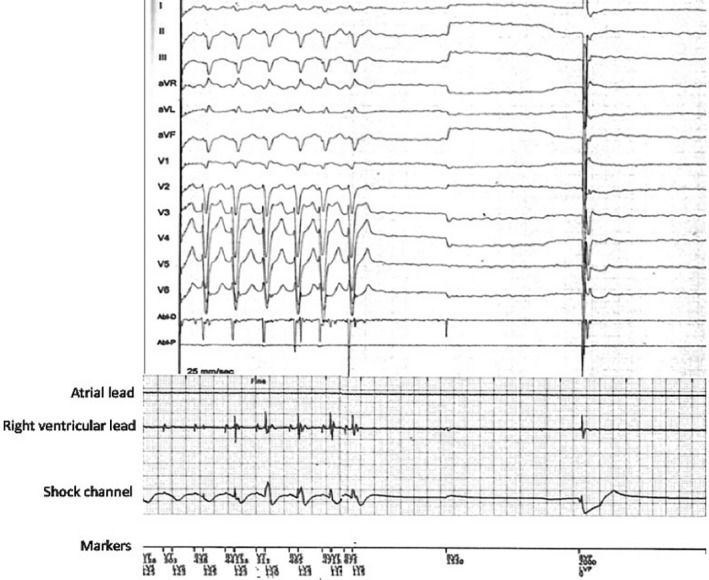
This picture is the most representative showing, in parallel, the 12‐lead electrocardiogram and intracavitary recordings from the ablation catheter (above) and the electrograms recorded by the CRT‐D during the radiofrequency application (below). Both irritative junctional tachycardia and overlapped external noise (sharp and big signals) can be observed. Then, after a pause, the CRT‐D was firstly inhibited by an apparent intrinsic potential sensed both by the distal dipole of the ablation catheter and by the device, then, 2 s later the device delivered a stimulus which was partially and locally captured

The deeper analysis of device electrograms revealed that the CRT‐D was first inhibited by an apparent intrinsic potential (Figures 1 and 2C,D—black, thin arrow) then after 2 seconds the device delivered a stimulus which likely was partially and locally captured (Figures 1 and 2C,D—black asterisk). Two seconds after another potential suppressed the CRT‐D (Figure 2D—red circle) and then an uncaptured pacing beat was observed.

**FIGURE 2:**
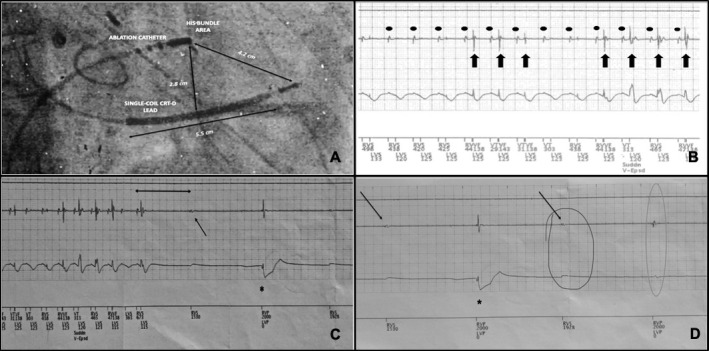
(A) A detail of the RAO projection showing the distance between the radiofrequency source and the CRT‐D lead (ranging from 2.8 to 4.2 cm). As a reference measure, the length of the CRT‐D coil (5.5 cm) has been shown. (B) A detail of the device's electrograms during the AV node ablation. From above to below, the following channels: atrial lead (switched off), right ventricular lead, shock channel, and markers. The black rounds point the junctional tachycardia during the RF application on the His bundle area. Black thick arrows show the persistence of an overlapped external noise which is later represented by bigger, distinct signals. (C) From above to below, the following channels: atrial lead (switched off), right ventricular lead, shock channel, and markers. First, the picture shows the combination of tachycardia and radiofrequency interference. Then, after the AV block a spontaneous signal (black, thin arrow) comes exactly after three cycle lengths from the last beat of the tachycardia (black double arrow). Later on, a stimulus which is probably partially and locally captured is represented by the black asterisk. (D) From above to below, the following channels: atrial lead (switched off), right ventricular lead, shock channel, and markers. From the left, a small, spontaneous potentials sensed by the device (thin, black arrow), and then, a pacing beat delivered by the device which was partially captured by the myocardium (black asterisk). Then, again the small, local potential sensed by the device (black, thin arrow, and red circle) followed by another pacing beat with ineffective myocardial capture (orange circle)

Was the source of these potentials artifacts, RF interference or spontaneous myocardial depolarizations?

The reproducibility and detection of these signals by both the device (Figure 2D) and EP recording system (Figure 1) ruled out the artifacts' hypothesis. The RF interference, on the contrary, was excluded by the fact that the potentials were sensed by the distal dipole of the ablation catheter. Here, intracavitary recordings showed a sharp potential compatible with a local junctional potential (Figures 1 and 2C,D). Indeed, these signals were not recorded in the proximal dipole of the ablation catheter (2.5 mm far) but, surprisingly, sensed by the lead implanted in the mid‐low septum of the right ventricle. Furthermore, the signals displayed a similar morphology of the junctional tachycardia and appeared to be related to the last cycle of the tachycardia occurring exactly three cycles following the last beat (Figure 2C). Altogether, these observations suggest that the origin of the potentials is spontaneous myocardial depolarization.

A further question is if RF did interfere with the functions of the device. The analysis of device's electrograms revealed at the beginning of the energy delivery a very short period of typical “noise” by electromagnetic interference, which resulted in a transient oversensing of the device (Figure 2B). The overlap of the external noise with the ablation‐related junctional tachycardia produced an uncommon distinct and sharp signal (Figure 2B) probably because of the noise‐filtering properties of the device.

Finally, why did the pace fail to capture the myocardium (Figure 2D—orange circle on the right)? Asynchronous pacing, pacing inhibition, loss of capture, power‐on‐reset, and rarely runaway pacing have been previously reported during RF ablation.^1^, ^2^, ^3^, ^4^, ^5^ Loss of capture is usually temporary during the procedure although a permanent failure has been reported,^3^, ^4^ and is not related to oversensing and/or “appropriate” inhibition of pacing. More probably, the permanent or intermittent capture failures are secondary to the interference of the capacitor with a strong continuous noise resulting in reduced pacing pulse amplitude. This mechanism was described as exit block in former reports^5^ and is consistent with the self‐protection automatism of the device against high energy noise. During thermal ablation, current flows from the conducting electrode toward a dispersive or ground electrode.^1^ RF ablation generates signal frequencies between 500 and 1000 kHz in a unipolar configuration.^1^ The close proximity (within 4 cm) of the ablation catheter to the permanent pacing lead seems to increase the risk of pacing dysfunctions for both pacemakers and defibrillators.^2^ In our case, the direct contact between the ablation catheter and the implanted CRT‐D lead was avoided during all the procedure. However, the distance between the RF source and the lead was 2.8‐4.2 cm (Figure 2A—black arrows), which, along with the large‐tip electrode of the ablation catheter, might easily explain the interference of RF on the ventricular lead channel.

The interference of RF ablation with cardiac electronic devices has been already extensively described and is not uncommon. Fully understanding the interactions between the cardiac devices and RF source is crucial for avoiding dysfunctions of the device as in this case and might be helpful in the future.

## DISCLOSURE

No conflicts of interest to be declared.
